# ABCC6 plays a significant role in the transport of nilotinib and dasatinib, and contributes to TKI resistance *in vitro*, in both cell lines and primary patient mononuclear cells

**DOI:** 10.1371/journal.pone.0192180

**Published:** 2018-01-31

**Authors:** Laura N. Eadie, Phuong Dang, Jarrad M. Goyne, Timothy P. Hughes, Deborah L. White

**Affiliations:** 1 Cancer Theme, South Australian Health and Medical Research Institute (SAHMRI), Adelaide, South Australia; 2 School of Medicine, Faculty of Health Sciences, University of Adelaide, Adelaide, South Australia; 3 Division of Haematology, SA Pathology, Adelaide, South Australia; 4 School of Paediatrics, Faculty of Health Sciences, University of Adelaide, Adelaide, South Australia; 5 School of Biological Sciences, Faculty of Sciences, University of Adelaide, Adelaide, South Australia; Istituto di Genetica Molecolare, ITALY

## Abstract

ATP Binding Cassette family efflux proteins ABCB1 and ABCG2 have previously been demonstrated to interact with Tyrosine Kinase Inhibitors (TKIs); however, evidence for the interaction of other potentially relevant drug transporters with TKIs is lacking. Through Taqman transporter array technology we assessed the impact of nilotinib on mRNA expression of ABC transporters, with ABCC6 identified as a transporter of interest. Additionally, increased expression of *ABCC6* mRNA was observed during *in vitro* development of nilotinib resistance in *BCR-ABL1*-expressing cell lines. K562 cells exposed to gradually increasing concentrations of nilotinib (to 2 μM) expressed up to 57-fold higher levels of *ABCC6* mRNA when compared with control cells (*p* = 0.002). Analogous results were observed in nilotinib resistant K562-Dox cells (up to 33-fold higher levels of *ABCC6*, *p* = 0.002). IC50 experiments were conducted on patient mononuclear cells in the absence and presence of three ABCC6 inhibitors: indomethacin, probenecid and pantoprazole. Results demonstrated that all three inhibitors significantly reduced nilotinib IC50 (*p*<0.001) indicating ABCC6 is likely involved in nilotinib transport. Cell line data confirmed these findings. Similar results were obtained for dasatinib, but not imatinib. Combined, these studies suggest that nilotinib and dasatinib are likely substrates of ABCC6 and to our knowledge, this is the first report of ABCC6 involvement in TKI transport. In addition, ABCC6 overexpression may also contribute to nilotinib and dasatinib resistance *in vitro*. With nilotinib and dasatinib now front line therapy options in the treatment of CML, concomitant administration of ABCC6 inhibitors may present an attractive option to enhance TKI efficacy.

## Introduction

Chronic Myeloid Leukaemia (CML) is caused by the Breakpoint cluster region-abelson (Bcr-Abl) oncoprotein, which is effectively inhibited by Tyrosine Kinase Inhibitors (TKIs) such as imatinib[1] and the second generation inhibitors nilotinib[2] and dasatinib[3]. Resistance to TKIs can occur due to numerous factors including Bcr-Abl overexpression and most commonly because of mutations in the Bcr-Abl kinase domain[4]. However, mounting evidence emphasises the importance of cellular drug efflux transporters, in particular members of the ATP-Binding Cassette (ABC) family, and attendant intracellular drug levels in TKI response. Indeed, recent data has highlighted P-glycoprotein (ABCB1) overexpression as a contributor to TKI resistance[5] and as a potential biomarker for predicting patient response to imatinib[6].

Members of the ABC family of transporters are responsible for the extrusion of small molecules, chemotherapeutics and other xenobiotics, including TKIs, from cells[7]. In particular, ABCB1 and breast cancer resistance protein (BCRP/ABCG2) have previously been implicated in multidrug resistance and their interaction with TKIs has been thoroughly investigated and reviewed[8]. More recently the role of ABCC1, ABCC3, ABCC4[9] and ABCA3[10] in TKI transport and resistance has also been examined. However, to date, no investigations into the TKI:ABCC6 relationship have been conducted.

ABCC6 (MRP6) belongs to the Multidrug Resistance Protein (MRP) superfamily and is capable of extruding a wide variety of substrates from cells[11]. ABCC6 bears striking sequence homology to ABCC1 and is thought to have arisen from a gene duplication[12–14]. While a biological substrate has not yet been elucidated, ABCC6 is known to transport lipophilic molecules of negative charge[15, 16] and is primarily expressed at points of drug extrusion such as the liver and the kidney[12, 17, 18]. The involvement of ABCC6 in an autosomal recessive connective tissue disorder, pseudoxanthoma elasticum, is well established despite the fact that ABCC6 is not expressed in the tissue within which this disease occurs[19, 20].

The involvement of ABCC6 in the transport of, and resistance to, a number of anticancer agents including etoposide, doxorubicin and daunorubicin has been demonstrated[15, 21]. However, ABCC6 involvement in the transport of other small molecule inhibitors such as TKIs has not been investigated. Our previous study[22] demonstrated a reduction in nilotinib IC50 (based on Bcr-Abl kinase inhibition) in ABCB1 overexpressing K562-Dox cells in the presence of proton pump inhibitors (PPI) known to inhibit ABCB1[23, 24]. The reduction observed suggests that PPIs block ABCB1-mediated efflux of nilotinib, increasing the intracellular concentration and subsequent kinase inhibition thus reducing the IC50. Intriguingly, the IC50 was also reduced in K562 cells, albeit to a lesser extent, which do not overexpress ABCB1[22]. These results suggest that an alternate transporter expressed in both K562-Dox and K562 cells may also be responsible for the efflux of nilotinib leading to the lowered IC50. PPIs have been previously demonstrated to inhibit ABCC6[15, 23, 25] and given the overlapping substrate specificities of ABC transporters, further investigations into the TKI:ABCC6 relationship were warranted. Here we provide the first evidence that ABCC6 may be involved in TKI transport and that overexpression of ABCC6 likely causes resistance to nilotinib *in vitro*.

## Materials and methods

### Ethics statement

This research involved human clinical samples and these correlative studies were approved by the Royal Adelaide Hospital Research Ethics Committee (RAH protocol number: 070718). All samples were collected with informed consent in accordance with the Institutional Ethics approved protocols and with reference to the Declaration of Helsinki.

### Cell lines

*BCR-ABL1*-expressing cell lines KU812 and K562 were obtained from American Type Culture Collection (ATCC; Manassas, VA). K562 cells were transfected as described previously and the resultant K562-ABCG2 cells cultured in 500 μg/mL G418 (Invitrogen, Carlsbad, CA) and assessed for appropriate ABCG2 expression by quantitative PCR and flow cytometry[26]. K562-Dox cells (ABCB1-overexpressing) were generated after the continuous culture of K562 parental cells in the ABCB1 substrate, doxorubicin. K562-Dox cells now stably express ABCB1 in the absence of doxorubicin (kindly provided by Prof. Leonie Ashman, University of Newcastle, Callaghan, NSW). HepG2 cells were kindly provided by Prof. Andrew Zannettino (South Australian Health and Medical Research Institute/University of Adelaide, Adelaide, SA). All suspension cells were cultured as described previously[27]; HepG2 cells were cultured in Dulbecco’s Modified Eagle Media (DMEM; Sigma). Prior to reculture (1:20 dilution), cells were trypsinised (0.25%; Sigma), separated by pipetting and resuspended in fresh media devoid of trypsin. Nilotinib-[5], dasatinib-[28] and imatinib-resistant[28] cell lines were generated as described previously. Briefly, cell lines were maintained in liquid culture and were gradually exposed to escalating concentrations of TKI. TKI concentrations were increased once cells demonstrated tolerance to the current concentration (>80% survival in culture for >10 days).

### Patient cells

Peripheral blood was obtained from *de novo* chronic phase CML patients before commencement of TKI therapy and mononuclear cells (MNCs) were isolated using Lymphoprep (Axis Shield, Oslo, Norway) density gradient centrifugation.

### TKIs and efflux transporter inhibitors

Imatinib mesylate (Glivec^®^) and nilotinib (Tasigna^®^) were provided by Novartis Pharmaceuticals (Basel, Switzerland), dasatinib (Sprycel^®^) was provided by Bristol-Myers Squibb (Victoria, Australia). Stock solutions of imatinib were prepared at 10 mM in distilled water, sterile filtered and stored at -80°C. Stock solutions of nilotinib and dasatinib were prepared at 10 mM in dimethylsulfoxide (DMSO; Sigma, St Louis, MO) and stored at 4°C. Verapamil (Royal Adelaide Hospital (RAH) Pharmacy) was used at 50 μM from a 2.5 mg/mL stock; pantoprazole (RAH Pharmacy) was used at 200 μM from a 10 mM stock; indomethacin (Sigma) was used at 100 μM from a 10 mg/mL stock; probenecid (Sigma) was used at 1 mM from a 175 mM stock; PSC-833 is a Cyclosporin A derivative kindly provided by Novartis Pharmaceuticals and was used at 10 μM from 8.23 mM stock. The concentrations of inhibitors were chosen based on specificity of ABC transporter inhibition and previous *in vitro* experimentation (S1 Table).

### p-CRKL determined IC50 and western blotting

*BCR-ABL1*-expressing cells (2×10^5^ cells) or 2×10^6^ patient mononuclear cells (MNCs) were incubated for 2 h at 37°C/5%CO_2_ with concentrations of nilotinib (NIL) ranging 0–100 μM, imatinib (IM) ranging 0–100 μM and dasatinib (DAS) ranging 0–5000 nM. Following incubation, cells were lysed in Laemmli’s buffer[29] before resolution by 12% SDS-PAGE and electrophoretic transfer to PVDF membrane (GE Healthcare, Buckinghamshire, UK) at 65 mA overnight. Western blotting for phosphorylated CT10 regulator of kinase like (p-CRKL) was performed as previously described[30]. IC50 values (IC50^NIL^, IC50^DAS^, IC50^IM^) were determined as the dose of drug required to reduce p-CRKL levels by 50% and are presented as mean ± SEM. Western blotting for ABCC6 was performed as described in Supplemental methods.

### Taqman^®^ array—Human drug transporters

The Taqman^®^ transporter arrays (Cat# 4414118; Thermo Fisher Scientific, Waltham, MA, USA) utilise gene-specific primer and probe sets in order to compare gene expression between treated and untreated samples. The arrays were carried out according to the manufacturer’s instructions.

#### Real time quantitative polymerase chain reaction (RQ-PCR)

1×10^7^ intermediately resistant cells (produced during TKI resistance generation[5]) were stored in TRIzol stabilization solution (Invitrogen Life Technologies) at -80°C. RNA was extracted using the phenol/chloroform method[31] and cDNA synthesized using random hexamers (GeneWorks, Hindmarsh, SA, Australia) and Superscript II reverse transcriptase (Invitrogen Life Technologies). Primers were designed using Primer Express software v2.0 (Applied Biosystems, Foster City, CA, USA), and sequences were as described in Supplemental methods. Amplification was performed using RT2 real-time SYBR Green/ROX PCR Master Mix (SuperArray Bioscience, Frederick, MD, USA) on a RotorGene real time PCR machine (Corbett Research, San Francisco, CA, USA). Results were analysed using Rotor-Gene 6000 Series software (Corbett Research) and the relative expression levels of the transporters were calculated by the comparative Ct method using the 2ΔΔCt formula to achieve results for relative quantification. The *ABCC6* control cell line HepG2 was used as a calibrator and all samples were normalized to the house keeping gene *BCR*.

### Statistics

Statistical tests were performed using the GraphPad Prism 6 statistical software (GraphPad Prism Inc, La Jolla, CA, USA). Normality tests were performed on each data set using the D’Agostino & Pearson omnibus normality test. The Mann-Whitney Rank Sum or the Student’s *t*-test were used to determine differences between experimental groups depending on whether the data sets failed or passed the normality test, respectively. Differences were considered to be statistically significant when the probability value (*p*-value) was <0.05.

## Results

### ABCB1 inhibition does not significantly affect nilotinib-mediated kinase inhibition in patient mononuclear cells

**Fig 1:**
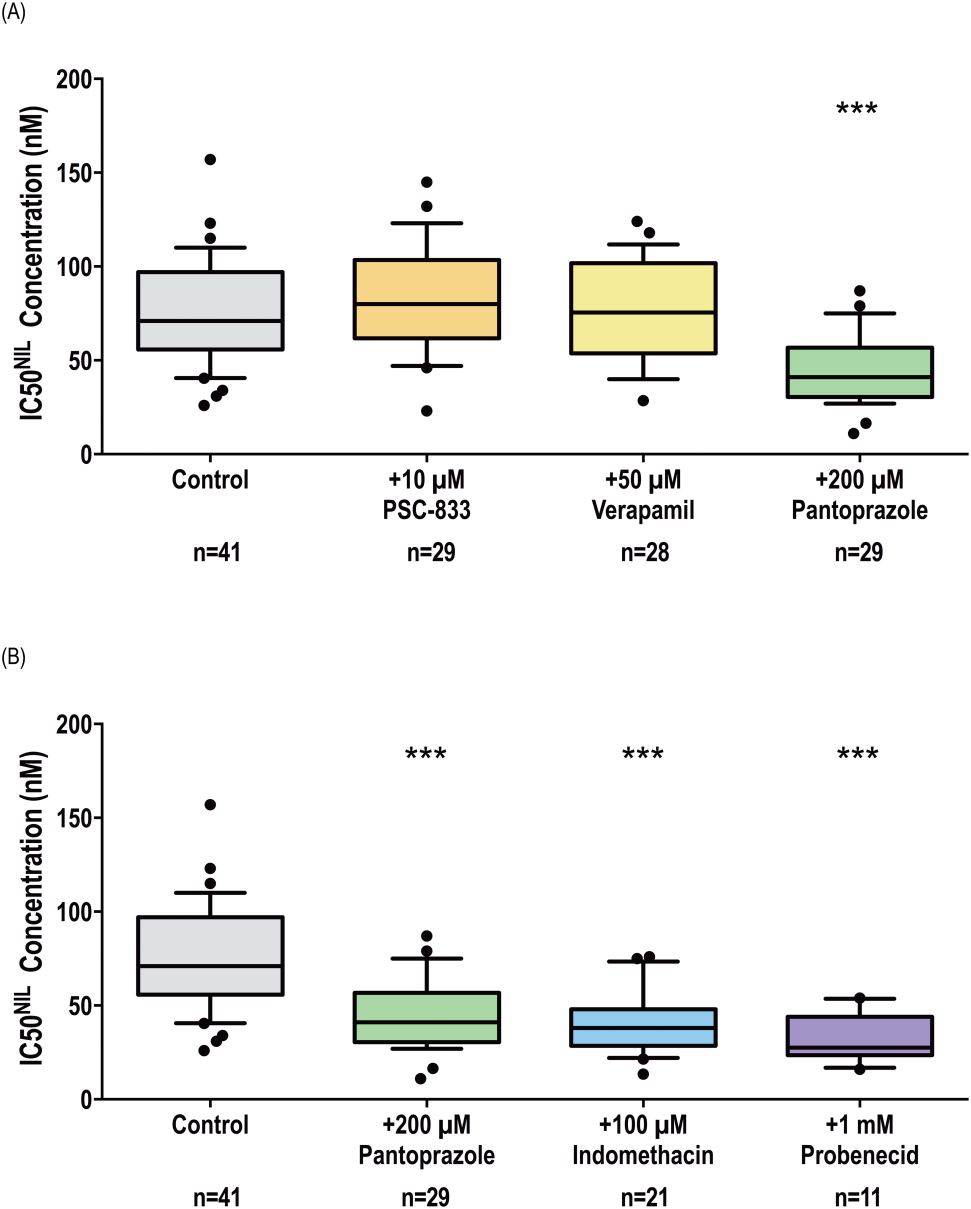
Chemical inhibition of ABCC6 but not ABCB1 increases the efficacy of nilotinib in patient MNCs. p-CRKL determined IC50^NIL^ was determined via incubating patient MNCs with increasing concentrations of nilotinib in the absence and presence of (A) three ABCB1 inhibitors: PSC-833, verapamil and pantoprazole and (B) three ABCC6 inhibitors: pantoprazole, indomethacin, probenecid. CRKL western blotting was performed to determine the concentration of nilotinib required for 50% Bcr-Abl kinase inhibition (IC50^NIL^). Representative western blots are shown in S1 Fig. The box plots depict the median, the upper 25th and the lower 75th percentiles while the whiskers encompass the 10th and 90th percentiles. Statistical analyses were performed using Student’s *t*-test with statistically significant *p*-values denoted by asterisks (*** p<0.001). NIL = nilotinib.

The prognostic value of monitoring early alterations in *ABCB1* mRNA expression levels in CML patient cells in order to predict patient response to imatinib has recently been described[6]. ABCB1 overexpression has also been implicated in nilotinib, imatinib and dasatinib resistance development *in vitro*[5]. For this reason, the effect of three ABCB1 inhibitors (pantoprazole, verapamil, PSC-833, S1 Table) on IC50^NIL^ was assessed in patient MNCs prior to the start of TKI therapy. The IC50 assay is a surrogate for sensitivity to Bcr-Abl kinase inhibition and it was expected that if ABCB1 is involved in the efflux of nilotinib from patient MNCs, inhibition of this transporter should increase concentrations of intracellular nilotinib resulting in a reduction in IC50^NIL^. Unexpectedly, a significant decrease in IC50^NIL^ was observed only in those cells incubated in the presence of 200 μM pantoprazole (Fig 1A, S1A Fig): 41 nM versus 71 nM in control cells cultured in the absence of inhibitors (*p*<0.001). The addition of 10 μM PSC-833 and 50 μM verapamil had no effect on IC50^NIL^: 76 nM and 80 nM respectively (*p*>0.05;).

### Nilotinib may be effluxed by other previously unidentified ABC transporters

We have also previously observed a significant decrease in IC50^NIL^ in *BCR-ABL1*+ cell lines in the presence of pantoprazole and to a lesser extent esomeprazole (dual ABCB1/ABCG2 inhibitors)[22]. Interestingly, comparable results were observed in two different cells lines regardless of ABCB1 expression: K562-Dox cells (ABCB1-overexpressing) and the parental K562 cells (negligible ABCB1/ABCG2). Thus, for the current study we investigated the effect of different concentrations of pantoprazole in a further two cell lines: KU812 (negligible ABCB1/ABCG2) and K562-ABCG2 cells (ABCG2-overexpressing)[27]. Results demonstrated a significant reduction in IC50^NIL^ in the presence of pantoprazole in all cell lines regardless of the ABCB1/ABCG2 expression status that was proportionate to the dose of pantoprazole (Table 1). The greatest reduction occurred in K562 cells (71% reduction in the presence of 500 μM pantoprazole, *p*<0.001), which express negligible levels of ABCB1 and ABCG2. These data, combined with observations in patient MNCs, suggest there may be other clinically relevant ABC efflux transporter/s that are involved in nilotinib transport and are inhibitable by pantoprazole.

**Table 1:** The effect of increasing concentrations of pantoprazole on IC50^NIL^ in *BCR-ABL1*+ cell lines.

Cell Line	Transporter Expression		IC50^NIL^ (nM)	Decrease (%)	*p*-value
	ABCB1	ABCG2			
K562					
Control (n = 6)	✘	✘	388		
+50 μM PP (n = 3)	✘	✘	254	34	*p* = 0.012
+200 μM PP (n = 5)	✘	✘	217	44	*p* = 0.002
+500 μM PP (n = 4)	✘	✘	114	71	*p* = 0.0002
K562-Dox					
Control (n = 5)	✔	✘	463		
+50 μM PP (n = 3)	✔	✘	202	56	*p* = 0.021
+200 μM PP (n = 4)	✔	✘	201	57	*p* = 0.010
+500 μM PP (n = 3)	✔	✘	145	69	*p* = 0.010
K562-ABCG2					
Control (n = 6)	✘	✔	261		
+50 μM PP (n = 5)	✘	✔	122	53	*p* = 0.007
+100 μM PP (n = 5)	✘	✔	157	40	*p* = 0.041
+200 μM PP (n = 5)	✘	✔	120	54	*p* = 0.011
KU812					
Control (n = 5)	✘	✘	305		
+50 μM PP (n = 5)	✘	✘	149	51	*p* = 0.010
+100 μM PP (n = 5)	✘	✘	146	52	*p* = 0.011
+250 μM PP (n = 5)	✘	✘	117	62	*p* = 0.004

Statistical analyses were performed using Student’s *t*-test; NIL = nilotinib, PP = pantoprazole.

### Exposure to nilotinib increases expression of ABCC6 *in vitro*

**Fig 2:**
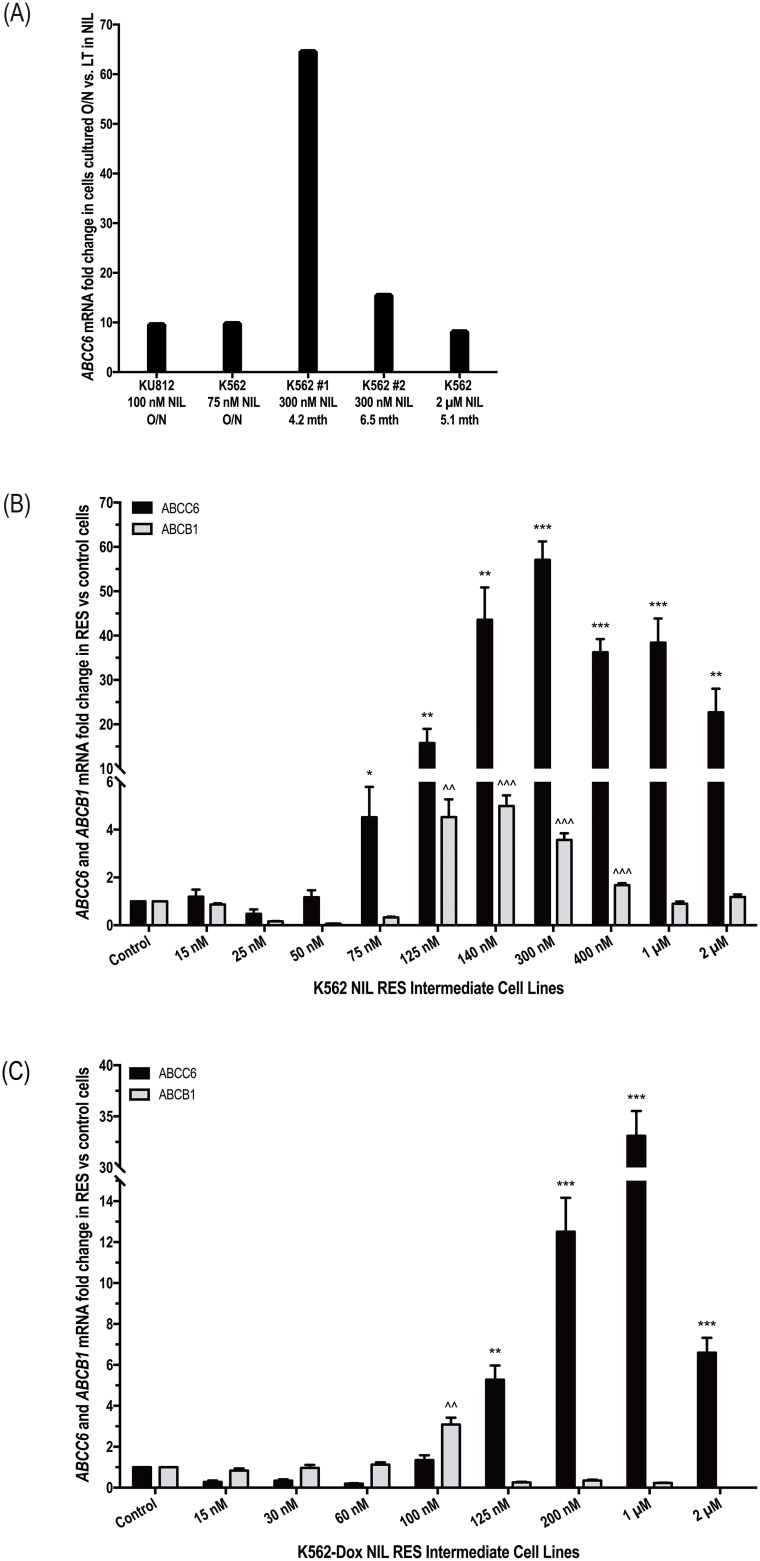
*ABCB1* and *ABCC6* mRNA levels increase in concert during development of nilotinib resistance in *BCR-ABL1*+ cell lines. (A) Expression levels of *ABCC6* mRNA were assessed by Taqman^®^ transporter array in K562 and KU812 cells exposed transiently (overnight, O/N) and long term to nilotinib Expression levels of *ABCC6* and *ABCB1* mRNA were assessed in (B) K562 and (C) K562-Dox cells gradually made resistant to nilotinib by exposure to increasing concentrations over time. (A) *ABCC6* levels were normalized to selected control genes (as determined by Thermo Fisher Scientific DataAssist Software v1.0) and fold change in cells cultured in the presence of nilotinib calculated relative to cells cultured in the absence of nilotinib. (B-C) *ABCC6* and *ABCB1* levels were normalized to the housekeeping gene *BCR* and fold change in resistance intermediates calculated relative to control cells (control cell fold change was set at 1). The mRNA expression represents the mean of six independent experiments performed in triplicate. Statistical analyses were performed using Student’s *t*-test. Statistically significant *p*-values are denoted by asterisks (ABCC6) and carets (ABCB1) * p<0.05; ** p<0.01; *** p<0.001). Error bars represent SEM. NIL = nilotinib; RES = resistant.

Previous studies have demonstrated that incubation with various cytotoxic agents results in increased mRNA expression of transporters known to be relevant for those drugs[32, 33]. Thus, in order to identify other candidate transporters potentially involved in the efflux of nilotinib, commercially available Taqman^®^ transporter array plates were used to assess mRNA expression levels in *BCR-ABL1+* K562 and KU812 cells incubated overnight in the absence and presence of 75 nM and 100 nM nilotinib respectively. Additionally, K562 cells that had been cultured long term in nilotinib[5] were also assessed for alterations in transporter expression compared with control cells (Fig 2A). Results demonstrated a consistent increase in *ABCC6* mRNA in response to nilotinib exposure, highlighting ABCC6 as a likely candidate for nilotinib transport. In K562 and KU812 cells exposed transiently to nilotinib, *ABCC6* mRNA levels were increased 9.7- and 9.5-fold respectively compared with cells incubated in the absence of nilotinib; in K562 cells exposed long term to 300 nM and 2 μM nilotinib, *ABCC6* mRNA levels increased up to 64-fold compared with control cells (Fig 2A). These results were validated through assessment of *ABCC6* mRNA levels over the course of nilotinib resistance generation in K562 and K562-Dox intermediately resistant cells[5]. *ABCC6* mRNA levels increased significantly at the onset of nilotinib resistance in both cell lines (Fig 2B and 2C). In K562 cells, levels peaked at 57-fold greater in the 300 nM NIL cells compared with control cells (*p* = 0.002) while in K562-Dox cells the 1 μM NIL cells demonstrated 33-fold greater *ABCC6* mRNA levels compared with control cells (*p* = 0.002).

### ABCC6 protein is expressed in *BCR-ABL1*+ cell lines and ABCC6 inhibition increases efficacy of nilotinib *in vitro*

**Fig 3:**
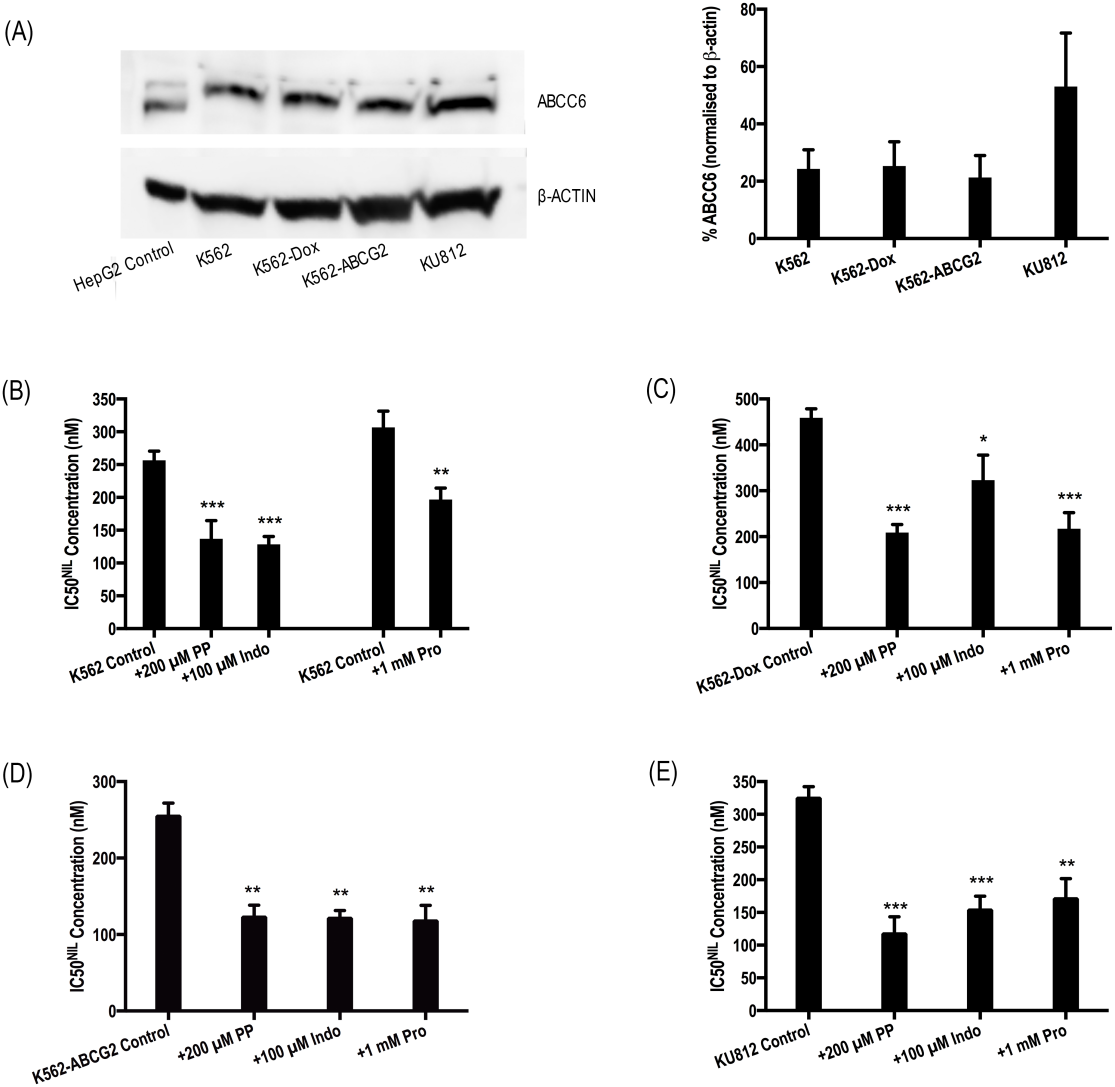
Chemical inhibition of ABCC6 increases the efficacy of nilotinib in *BCR-ABL1*+ cell lines. (A) Expression levels of ABCC6 protein were assessed in K562, K562-Dox, K562-ABCG2 and KU812 *BCR-ABL1*+ cell lines. The western blot analyses are representative and the corresponding densitometry analysis represents the mean of three experiments. HepG2 cells were used as a positive control and protein levels were normalised to β-ACTIN. HepG2 cells express two splice variants of ABCC6; *BCR-ABL1*+ cell lines express the N-glycosylated isoform only. (B) K562 (C) K562-Dox (D) K562-ABCG2 and (E) KU812 cells were incubated with increasing concentrations of nilotinib in the absence and presence of three ABCC6 inhibitors (200 μM pantoprazole, 100 μM indomethacin, 1 mM probenecid). CRKL western blotting was performed to determine the concentration of TKI required for 50% Bcr-Abl kinase inhibition (IC50^NIL^). Two K562 control bars are shown as experiments in the two groups were performed months apart. Error bars represent SEM. Representative western blots are shown in S2 Fig. NIL = nilotinib; PP = pantoprazole; Indo = Indomethacin; Pro = probenecid.

Before ABCC6 could be validated as a likely transporter of nilotinib, confirmation was required of a) ABCC6 expression in *BCR-ABL1*+ cell lines and b) pantoprazole-mediated inhibition of ABCC6. Indeed, all four cell lines expressed detectable levels of ABCC6 (Fig 3A) and a comprehensive literature search for inhibitor cross reactivity revealed pantoprazole likely inhibits ABCC6 (S1 Table). Thus, the ABCC6:TKI interaction warranted further investigation in cell lines and patient MNCs. Confoundingly, promiscuity in transporter inhibition for drugs such as pantoprazole make it difficult to isolate the transporter upon which a given inhibitor is acting. For this reason, a panel of ABCC6 inhibitors, with overlapping inhibition profiles, was used to ascertain the involvement of ABCC6 in the transport of nilotinib. This approach that has been successfully employed previously to eliminate transporters unlikely to interact with imatinib[34].

IC50^NIL^ experiments were repeated in the presence of pantoprazole as well as two additional ABCC6 inhibitors: indomethacin and probenecid. The addition of all inhibitors significantly reduced IC50^NIL^ in all cell lines studied (Fig 3B–3D, S2 Fig). In KU812 cells: IC50^NIL^ was reduced from 325 nM to 117 nM (*p*<0.001), to 154 nM (*p*<0.01) and to 171 nM (*p*<0.05) in the presence of pantoprazole, indomethacin and probenecid respectively. Similar results were observed in K562, K562-Dox and K562-ABCG2 cells. The relevance of ABCC6-mediated transport of nilotinib was then investigated in the patient setting (Fig 1B, S1B Fig). Importantly, when patient MNCs were cultured in the presence of indomethacin or probenecid, a reduction in IC50^NIL^ was observed, confirming observations made in the presence of pantoprazole as well as those made in cell lines. ABCC6 inhibition reduced IC50^NIL^ from 71 nM in cells incubated in the absence of inhibitors to 38 nM and 28 nM in cells cultured with indomethacin and probenecid respectively (*p*<0.001). Taken together, these results indicate the likely role of ABCC6 in the transport of nilotinib.

### ABCC6 inhibition increases the efficacy of dasatinib, but not imatinib, in both BCR-ABL1+ cell lines and patient MNCs

**Fig 4:**
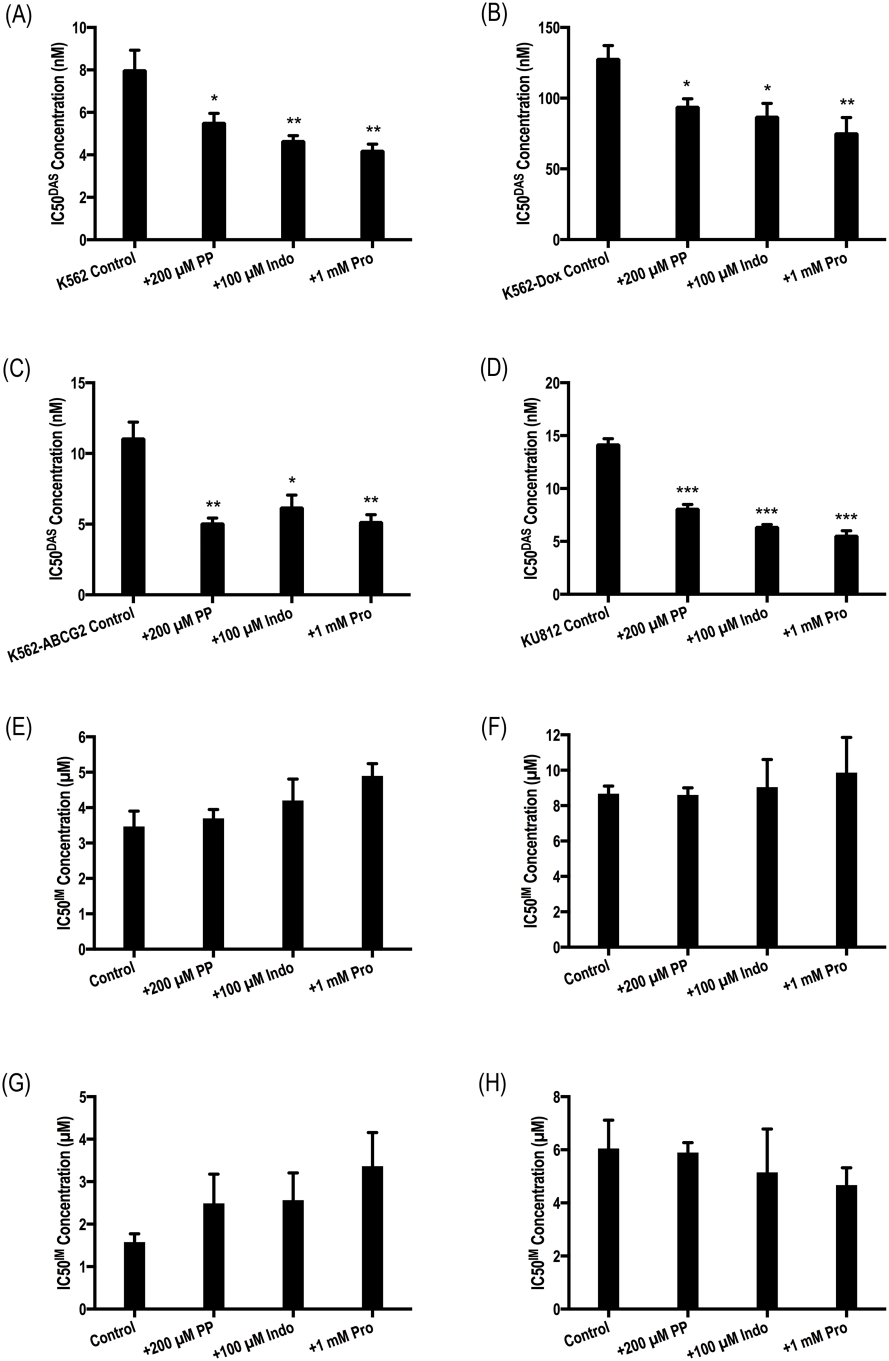
Chemical inhibition of ABCC6 increases the efficacy of dasatinib but not imatinib in *BCR-ABL1*+ cell lines. (A,E) K562 (B,F) K562-Dox (C,G) K562-ABCG2 and (D,H) KU812 cells were incubated with the indicated concentrations of (A-D) dasatinib and (E-H) imatinib in the absence and presence of three ABCC6 inhibitors (200 μM pantoprazole, 100 μM indomethacin, 1 mM probenecid). CRKL western blotting was performed to determine the concentration of TKI required for 50% Bcr-Abl kinase inhibition (IC50). Error bars represent SEM. Representative western blots are shown in S3 and S4 Figs. IM = imatinib; DAS = dasatinib; PP = pantoprazole; Indo = Indomethacin; Pro = probenecid.

**Fig 5:**
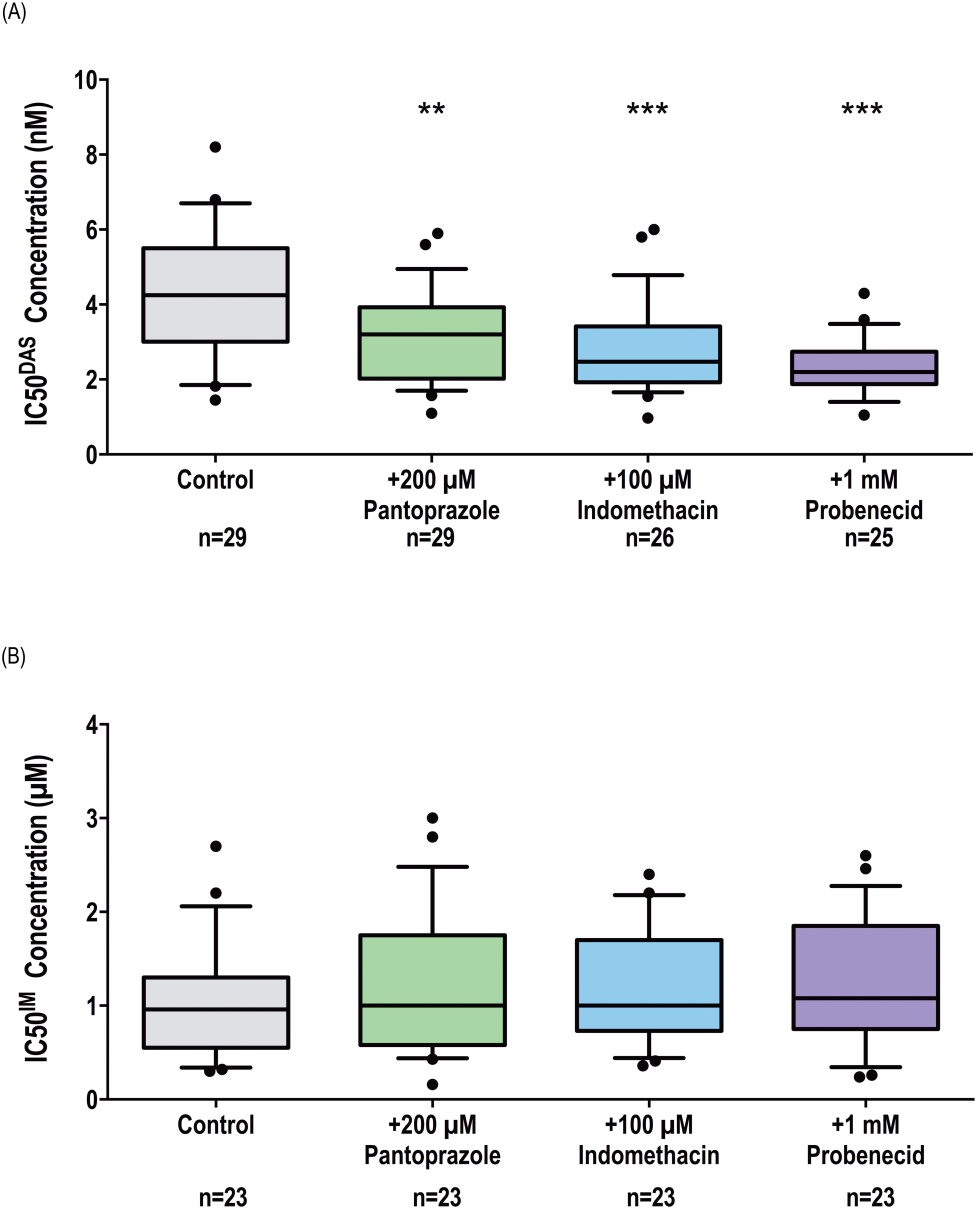
Chemical inhibition of ABCC6 increases the efficacy of dasatinib but not imatinib in patient MNCs. p-CRKL determined IC50 was determined via incubating patient MNCs with increasing concentrations of (A) dasatinib and (B) imatinib in the absence and presence of three ABCC6 inhibitors: pantoprazole, indomethacin, probenecid. CRKL western blotting was performed to determine the concentration of TKI required for 50% Bcr-Abl kinase inhibition (IC50). Representative western blots are shown in S3 and S4 Figs. The box plots depict the median, the upper 25th and the lower 75th percentiles while the whiskers encompass the 10th and 90th percentiles. Statistical analyses were performed using Student’s *t*-test with statistically *p*-values denoted by asterisks (** *p*<0.01; *** *p*<0.001). IM = imatinib. DAS = dasatinib.

Analogous experiments testing the effect of ABCC6 inhibitors on the efficacy of dasatinib and imatinib were performed in *BCR-ABL1+* cell lines. Data demonstrated that the addition of pantoprazole, indomethacin or probenecid resulted in a significant reduction in IC50^DAS^ in all cell lines tested (*p*<0.05; Fig 4A–4D). No effect of ABCC6 inhibitors on IC50^IM^ was observed (Fig 4E and 4F) denoting imatinib as an unlikely substrate for ABCC6. The role of ABCC6 in the efflux of dasatinib and imatinib from CML patient MNCs was also investigated (Fig 5A, S5A Fig). Again, IC50 experiments were performed in the absence and presence of pantoprazole, indomethacin and probenecid. Results confirmed those observed in cell lines and demonstrated a significant decrease in IC50^DAS^ in the presence of all three inhibitors: IC50^DAS^ was reduced from 4.3 nM in the absence of inhibitor to 3.2 nM (*p* = 0.014), 2.5 nM (*p*<0.001) and 2.2 nM (*p*<0.001) in the presence of pantoprazole, indomethacin and probenecid respectively. In contrast, equivalent experiments investigating the effect of ABCC6 inhibition on imatinib efficacy in CML patient MNCs demonstrated no change in IC50^IM^ in the absence or presence of ABCC6 inhibition (Fig 5B, S5B Fig): IC50^IM^ in the absence of inhibitor = 0.96 μM versus 1 μM, 1 μM and 1.1 μM (*p*>0.05) in the presence of pantoprazole, indomethacin and probenecid respectively. Thus, while ABCC6 inhibition significantly increases the efficacy of dasatinib presumably through reduced dasatinib efflux, it is unlikely ABCC6 plays a role in the transport of imatinib in patient MNCs.

### ABCC6 is the most important transporter in development of nilotinib resistance and contributes to dasatinib resistance *in vitro*

We have previously examined *ABCB1* mRNA expression in cells made resistant to nilotinib[5] and now we compare this with *ABCC6* mRNA expression. Data suggest that ABCB1 and ABCC6 work in concert during the development of nilotinib resistance (Fig 2). Interestingly, ABCC6 overexpression appears to contribute to nilotinib resistance to a greater extent than ABCB1. In K562 cells, *ABCC6* expression levels increased in concordance with exposure to increasing nilotinib concentrations (up to 57-fold higher than expression levels in control cells, *p*<0.001) and remained high for the duration of nilotinib dose escalation (Fig 2B). In contrast, while *ABCB1* levels increased initially, 5-fold greater levels were observed in cells resistant to 140 nM nilotinib compared with control cells (*p*<0.001), continued exposure to escalating nilotinib concentrations resulted in reduction of *ABCB1* to levels comparable with those observed in control cells (Fig 2B). Similar results were observed during the generation of nilotinib resistance in K562-Dox cells (Fig 2C). Comparable results were obtained for dasatinib resistant cells but not imatinib resistant cells supporting IC50 data and a dasatinib:ABCC6 interaction (S6 Fig).

## Discussion

ABCC6 is primarily expressed in the liver and kidney[12, 17, 18] and confers resistance to a number of anti-cancer agents[15]. While it presents a likely and novel candidate to function as a TKI transporter, the ABCC6:TKI relationship has not previously been studied. Here we demonstrate for the first time, that ABCC6 plays a role in the export of nilotinib and dasatinib from CML patient MNCs and that ABCC6 inhibition increases the efficacy of these TKIs. Importantly, ABCC6 inhibition had no effect on imatinib efficacy indicating an unlikely role for this transporter in imatinib efflux and confirming the specificity of the ABCC6:nilotinib/dasatinib interaction. ABCC6 is known to transport substrates anionic in nature[35]. Imatinib predominantly exists *in vivo* as a cation owing to the large degree of protonation at physiological pH[36]. In contrast, nilotinib and dasatinib exist more often as anions and nilotinib is transported by a number of Organic Anion Transporters (OATs)[37]. These observations make nilotinib and dasatinib likely ABCC6 substrates, a notion supported by the data presented here.

ABCB1 has previously been implicated as an important transporter of TKIs in *BCR-ABL1*+ cell lines[8] and increases in expression during development of resistance to nilotinib have been observed *in vitro*[5, 38, 39]. In this study we investigated the impact of ABCB1 inhibitors on nilotinib-mediated Bcr-Abl kinase inhibition (IC50^NIL^). If nilotinib is indeed transported by ABCB1 one would expect the IC50^NIL^ to decrease in the presence of ABCB1 inhibition. However, results were unexpected with only pantoprazole demonstrating a significant effect on IC50^NIL^ in patient MNCs. There are two possible explanations for this; firstly, nilotinib is not transported by ABCB1, however, this seems unlikely given the large volume of *in vitro* data supporting ABCB1 transport. Alternatively, ABCB1 may not be a relevant transporter in patient MNCs. This hypothesis is more plausible given the low expression levels of ABCB1 protein our laboratory has observed in routine screening of *de novo* CML patients (unpublished data). However, high expression of ABCB1 has been demonstrated in primitive CD34+CD38– and CD34+CD38+ CML cells[40] as well as cells at the blood-brain barrier[41]. Thus, ABCB1-mediated TKI efflux may predominantly occur in primitive subsets of cells and in cells at blood-tissue barriers (CNS, brain, testes) enhancing exclusion of TKIs and providing a reservoir of residual leukaemic cells. In contrast, ABCC6-mediated transport of nilotinib may be more relevant in the MNC populations present in patients prior to receiving TKI therapy.

In addition to providing evidence that ABCC6 is the predominant transporter of nilotinib in patient MNCs, this study also suggests that ABCC6 is the most important transporter involved in development of nilotinib resistance. Previous studies have demonstrated that, upon exposure to certain xenobiotics, cells increase mRNA expression of transporters known to interact with the substrate under investigation[32, 33, 42–44]. *ABCC6* mRNA expression was investigated in two *BCR-ABL1*+ cell lines during development of nilotinib resistance *in vitro* with expression increasing concomitantly upon exposure to escalating nilotinib concentrations and remaining high. In contrast, increased ABCB1 expression appears to provide the initial platform required for development of additional mechanisms of resistance to TKIs *in vitro*, including the overexpression of ABCC6 presented here. This hypothesis is supported by previous research in both cell lines[5] and primary patient cells[6]. A similar pattern of transporter expression was observed in *BCR-ABL1*+ cell lines made resistant to dasatinib where increased *ABCB1* was followed by persistent overexpression of *ABCC6*. Increases in *ABCC6* expression did not occur during development of imatinib resistance in the cell line models here.

The results presented here offer an explanation for the clinical observation that the concomitant use of a PPI such as pantoprazole results in a better response to nilotinib in CML patients[45]. Those patients receiving the standard 300 mg twice daily (BID) nilotinib therapy who also received a PPI for >50% of their time undergoing nilotinib therapy demonstrated higher rates of Major Molecular Response (MMR, <0.1% *BCR-ABL1*^IS^) at 12 months compared with those patients who received nilotinib alone. The difference in MMR rates was even greater in patients receiving a 400 mg BID dose. While superior rates of MMR were observed in both newly diagnosed patients as well as imatinib-resistant or–intolerant patients receiving PPI co-treatment, the differences failed to reach statistical significance. This was likely due to the small sample sizes in each of the patient cohorts. Importantly, our data support those findings and provide justification for a standardized trial comparing the long-term responses to nilotinib with and without co-administration of an ABCC6 inhibitor such as pantoprazole.

Taken together these data support ABCC6-mediated transport of nilotinib and dasatinib in cell lines as well as in patient MNCs. *In vitro* evidence also suggests that ABCC6 overexpression may play a role in development of resistance to both nilotinib and dasatinib although the contribution to resistance in the clinical setting remains to be elucidated. Concomitant administration of inhibitors of ABC transporters have previously been demonstrated to increase the efficacy of various cytotoxic agents *in vitro*[46, 47]. Importantly, simultaneous administration of pantoprazole in patients receiving nilotinib therapy has been investigated with no adverse effect on TKI efficacy described[22, 45]; indeed increased rates of MMR were observed. In conclusion, our data suggest that ABCC6 functions as a novel efflux transporter of nilotinib and dasatinib, but not imatinib in *BCR-ABL*+ cell lines as well as patient MNCs. The difference in ABCC6 affinity we observed between first and second generation inhibitors could potentially be due to the cationic and anionic forms in which imatinib and nilotinib/dasatinib exist at physiological pH. Finally, inhibition of ABCC6 significantly decreases IC50^NIL^ and IC50^DAS^ in patient MNCs, most likely due to an increase in intracellular TKI concentrations. The data presented here provide a strong rationale for a trial investigating the concomitant use of pantoprazole with nilotinib and dasatinib in the treatment of *de novo* CP-CML patients to enhance the retention of these TKIs in the target MNC population.

## Supporting information

S1 Fig Chemical inhibition of ABCC6 but not ABCB1 increases the efficacy of nilotinib in patient MNCs. p-CRKL determined IC50^NIL^ was determined via incubating patient MNCs with the indicated concentrations of nilotinib in the absence and presence of a) three ABCB1 inhibitors: PSC-833, verapamil and pantoprazole and b) three ABCC6 inhibitors: pantoprazole, indomethacin, probenecid. CRKL western blotting was performed to determine the concentration of nilotinib required for 50% Bcr-Abl kinase inhibition (IC50^NIL^). The western blots depict one patient and are representative of typical results. NIL = nilotinib; Ver = verapamil; PP = pantoprazole; Indo = Indomethacin; Pro = probenecid.
(TIF)

S2 Fig Chemical inhibition of ABCC6 increases the efficacy of nilotinib in *BCR-ABL1*+ cell lines. p-CRKL determined IC50^NIL^ was determined via incubating a) K562 b) K562-Dox c) K562-ABCG2 and d) KU812 cells with the indicated concentrations of nilotinib in the absence and presence of three ABCC6 inhibitors (200 μM pantoprazole, 100 μM indomethacin, 1 mM probenecid). CRKL western blotting was performed to determine the concentration of TKI required for 50% Bcr-Abl kinase inhibition (IC50^NIL^). The western blots are representative of typical results. The arrows depict approximate nilotinib concentration required for achievement of equal levels of CRKL and p-CRKL proteins. NIL = nilotinib. PP = pantoprazole; Indo = Indomethacin; Pro = probenecid.
(TIF)

S3 Fig Chemical inhibition of ABCC6 increases the efficacy of dasatinib in *BCR-ABL1*+ cell lines. p-CRKL determined IC50^DAS^ was determined via incubating a) K562 b) K562-Dox c) K562-ABCG2 and d) KU812 cells with the indicated concentrations of dasatinib in the absence and presence of three ABCC6 inhibitors (200 μM pantoprazole, 100 μM indomethacin, 1 mM probenecid). CRKL western blotting was performed to determine the concentration of TKI required for 50% Bcr-Abl kinase inhibition (IC50^DAS^). The western blots are representative of typical results. The arrows depict approximate dasatinib concentration required for achievement of equal levels of CRKL and p-CRKL proteins. DAS = dasatinib; PP = pantoprazole; Indo = Indomethacin; Pro = probenecid.
(TIF)

S4 Fig Chemical inhibition of ABCC6 has no effect on the efficacy of imatinib in *BCR-ABL1*+ cell lines. p-CRKL determined IC50^IM^ was determined via incubating a) K562 b) K562-Dox c) K562-ABCG2 and d) KU812 cells with the indicated concentrations of imatinib in the absence and presence of three ABCC6 inhibitors (200 μM pantoprazole, 100 μM indomethacin, 1 mM probenecid). CRKL western blotting was performed to determine the concentration of TKI required for 50% Bcr-Abl kinase inhibition (IC50^IM^). The western blot analyses are representative of typical results. The arrows depict approximate imatinib concentration required for achievement of equal levels of CRKL and p-CRKL proteins. IM = imatinib; PP = pantoprazole; Indo = Indomethacin; Pro = probenecid.
(TIF)

S5 Fig Chemical inhibition of ABCC6 increases the efficacy of dasatinib but not imatinib in patient MNCs. p-CRKL determined IC50^IM^ and IC50^DAS^ were determined via incubating patient MNCs with the indicated concentrations of a) dasatinib and b) imatinib in the absence and presence of three ABCC6 inhibitors: pantoprazole, indomethacin, probenecid. CRKL western blotting was performed to determine the concentration of imatinib or dasatinib required for 50% Bcr-Abl kinase inhibition (IC50). The western blots depict one patient and are representative of typical results. DAS = dasatinib; IM = imatinib; PP = pantoprazole; Indo = Indomethacin; Pro = probenecid.
(TIF)

S6 Fig *ABCB1* and *ABCC6* mRNA levels increase in concert during development of dasatinib resistance in *BCR-ABL1*+ cell lines. Expression levels of *ABCC6* and *ABCB1* mRNA were assessed in a) K562 b-c) K562-Dox cells and c) KU812 gradually made resistant to a-b) dasatinib or c-d) imatinib by exposure to increasing concentrations of TKI over time. *ABCC6* and *ABCB1* levels were normalized to the housekeeping gene *GUSB* and fold change in resistance intermediates calculated relative to control cells (control cell fold change was set at 1). The mRNA expression represents a single experiment performed in triplicate. DAS = dasatinib; IM = imatinib; RES = resistant.
(TIF)
