# Correction: ABCC6 plays a significant role in the transport of nilotinib and dasatinib, and contributes to TKI resistance *in vitro*, in both cell lines and primary patient mononuclear cells

**DOI:** 10.1371/journal.pone.0203583

**Published:** 2018-08-31

**Authors:** The PLOS ONE Staff

S1 Table is omitted from the list of Supporting Information. It can be viewed below.

S1 File is also omitted from the list of Supporting Information. It can be viewed below.

The publisher apologizes for the errors.

## Supporting information

S1 Table Summary of inhibitors used in this study and the corresponding cellular transporters upon which they act. (DOCX)

S1 File Supplementary methods. (DOCX)
