# Supplementary figures and images for: ABCC6 plays a significant role in the transport of nilotinib and dasatinib, and contributes to TKI resistance in vitro, in both cell lines and primary patient mononuclear cells
Source: PLoS One. 2018 Jan 31;13(1):e0192180. doi: 10.1371/journal.pone.0192180 (PMC5792028; doi:10.1371/journal.pone.0192180)

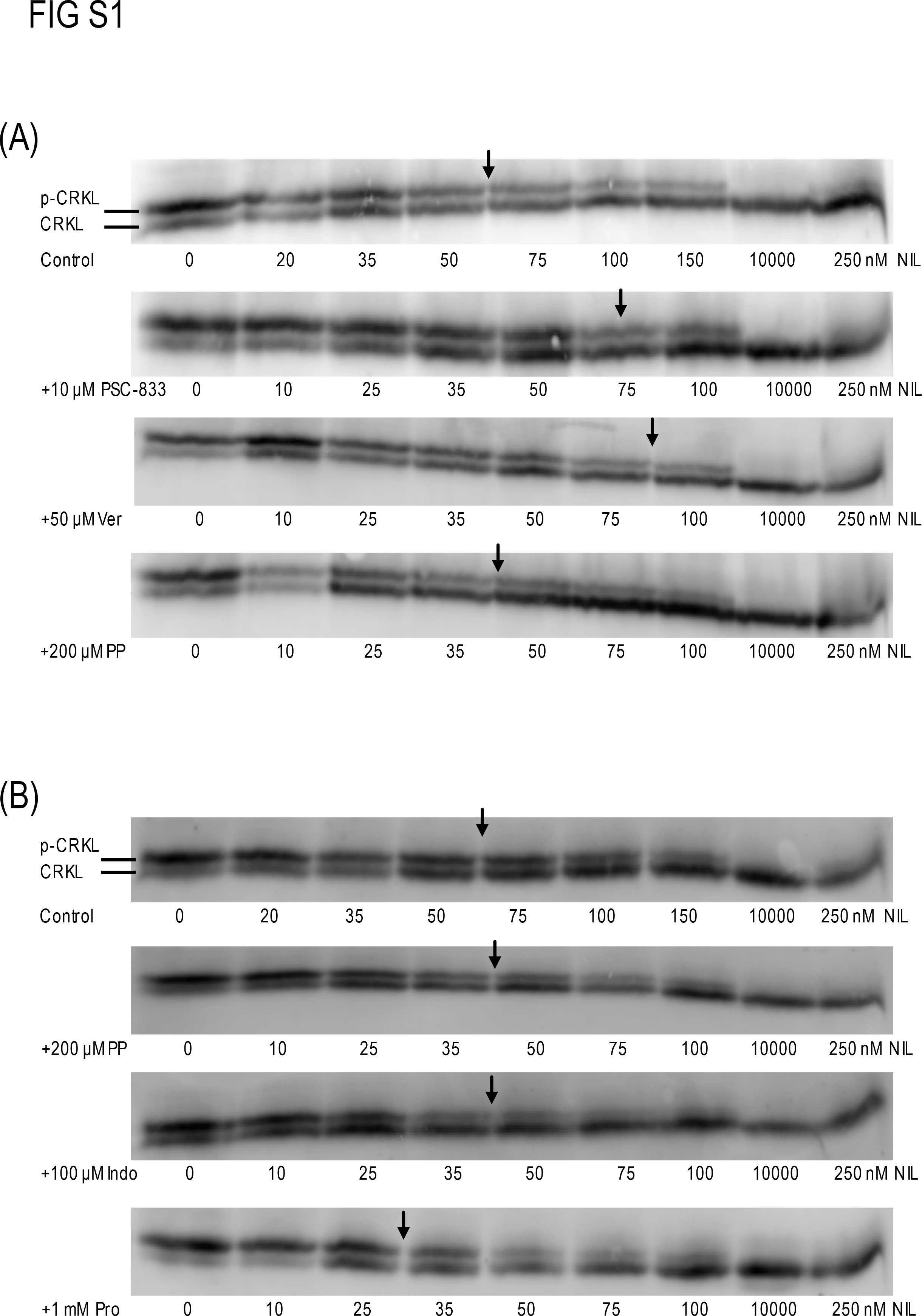

Supplement: S1 Fig — p-CRKL determined IC50NIL was determined via incubating patient MNCs with the indicated concentrations of nilotinib in the absence and presence of a) three ABCB1 inhibitors: PSC-833, verapamil and pantoprazole and b) three ABCC6 inhibitors: pantoprazole, indomethacin, probenecid. CRKL western blotting was performed to determine the concentration of nilotinib required for 50% Bcr-Abl kinase inhibition (IC50NIL). The western blots depict one patient and are representative of typical results. NIL = nilotinib; Ver = verapamil; PP = pantoprazole; Indo = Indomethacin; Pro = probenecid. (TIF) [file pone.0192180.s001.tif]

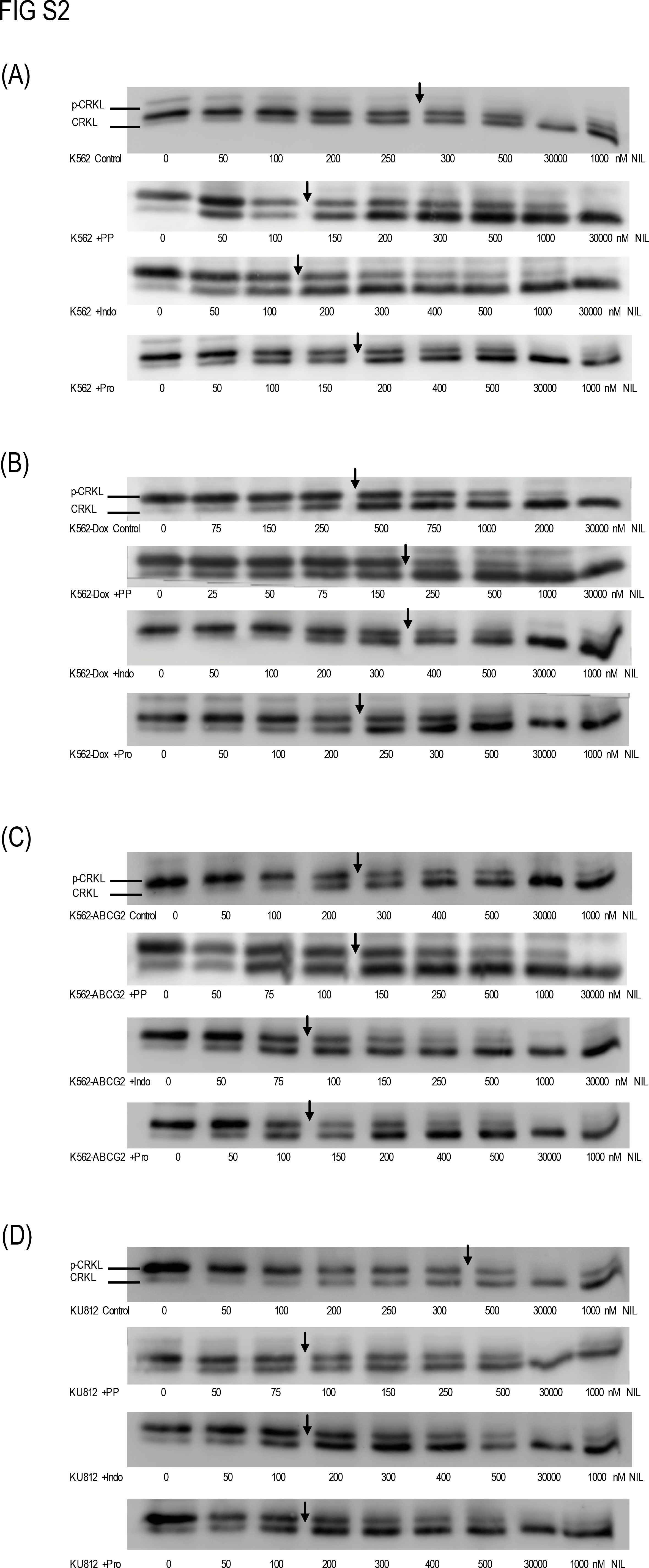

Supplement: S2 Fig — p-CRKL determined IC50NIL was determined via incubating a) K562 b) K562-Dox c) K562-ABCG2 and d) KU812 cells with the indicated concentrations of nilotinib in the absence and presence of three ABCC6 inhibitors (200 μM pantoprazole, 100 μM indomethacin, 1 mM probenecid). CRKL western blotting was performed to determine the concentration of TKI required for 50% Bcr-Abl kinase inhibition (IC50NIL). The western blots are representative of typical results. The arrows depict approximate nilotinib concentration required for achievement of equal levels of CRKL and p-CRKL proteins. NIL = nilotinib. PP = pantoprazole; Indo = Indomethacin; Pro = probenecid. (TIF) [file pone.0192180.s002.tif]

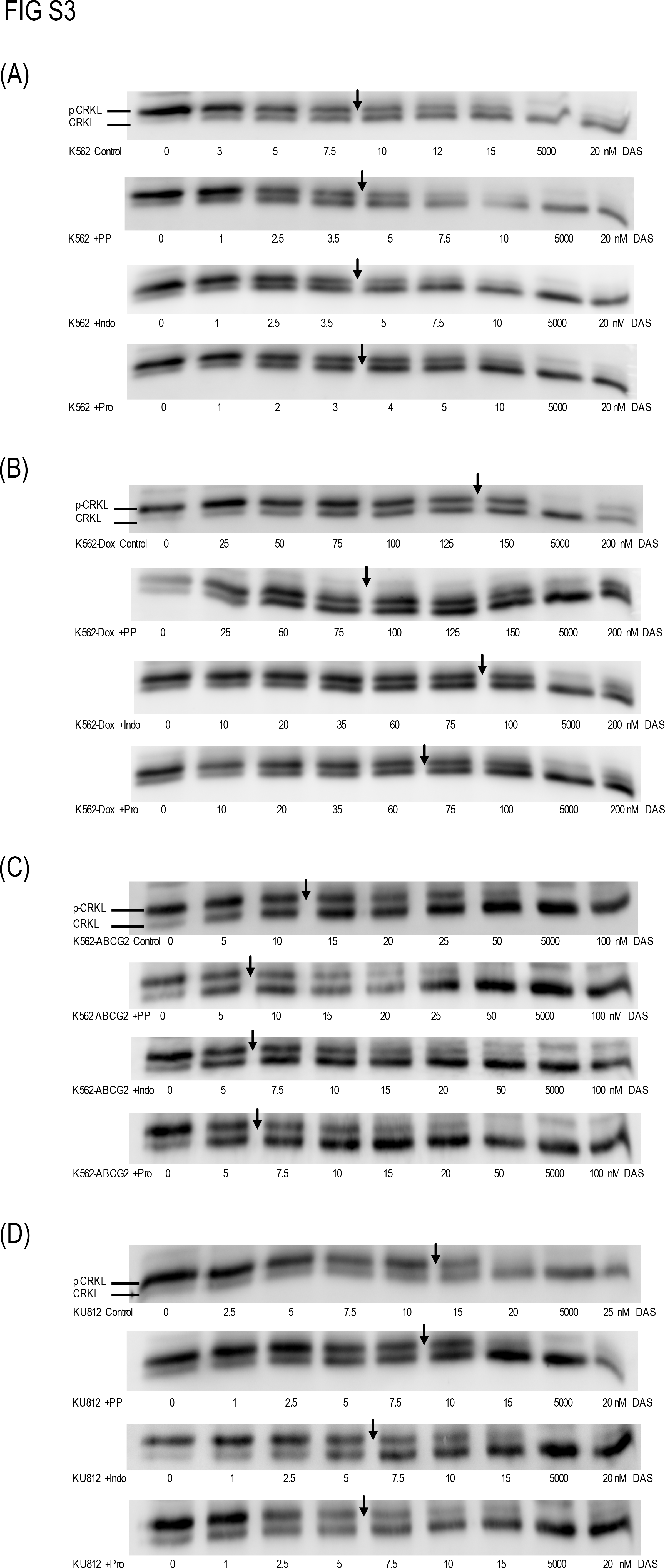

Supplement: S3 Fig — p-CRKL determined IC50DAS was determined via incubating a) K562 b) K562-Dox c) K562-ABCG2 and d) KU812 cells with the indicated concentrations of dasatinib in the absence and presence of three ABCC6 inhibitors (200 μM pantoprazole, 100 μM indomethacin, 1 mM probenecid). CRKL western blotting was performed to determine the concentration of TKI required for 50% Bcr-Abl kinase inhibition (IC50DAS). The western blots are representative of typical results. The arrows depict approximate dasatinib concentration required for achievement of equal levels of CRKL and p-CRKL proteins. DAS = dasatinib; PP = pantoprazole; Indo = Indomethacin; Pro = probenecid. (TIF) [file pone.0192180.s003.tif]

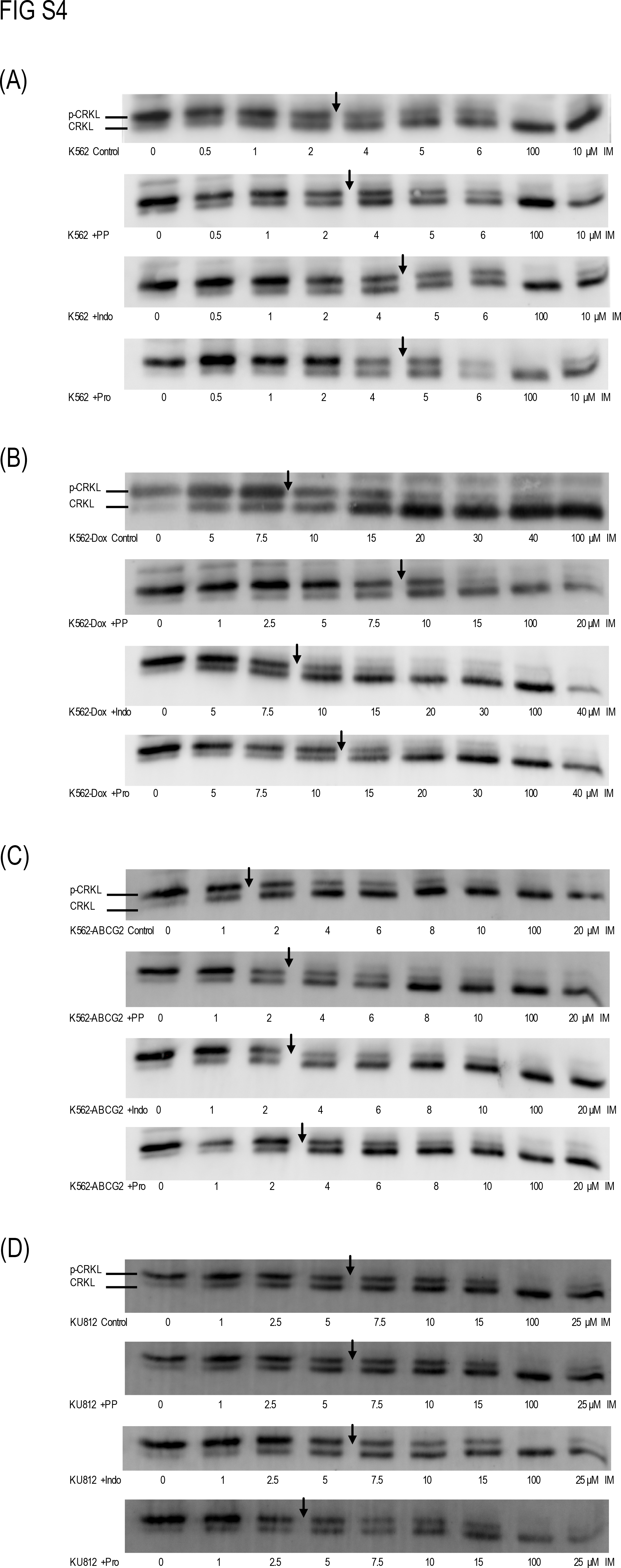

Supplement: S4 Fig — p-CRKL determined IC50IM was determined via incubating a) K562 b) K562-Dox c) K562-ABCG2 and d) KU812 cells with the indicated concentrations of imatinib in the absence and presence of three ABCC6 inhibitors (200 μM pantoprazole, 100 μM indomethacin, 1 mM probenecid). CRKL western blotting was performed to determine the concentration of TKI required for 50% Bcr-Abl kinase inhibition (IC50IM). The western blot analyses are representative of typical results. The arrows depict approximate imatinib concentration required for achievement of equal levels of CRKL and p-CRKL proteins. IM = imatinib; PP = pantoprazole; Indo = Indomethacin; Pro = probenecid. (TIF) [file pone.0192180.s004.tif]

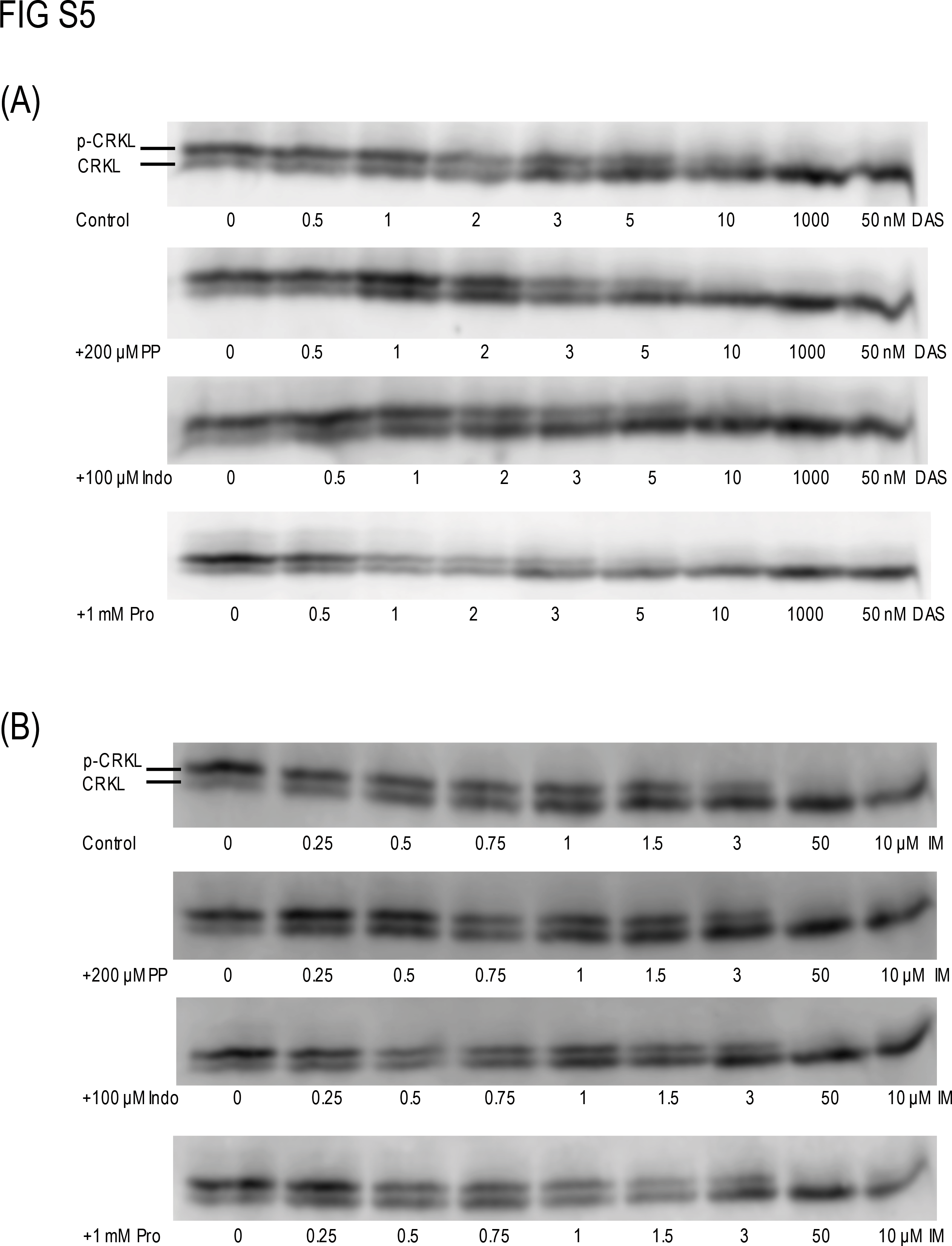

Supplement: S5 Fig — p-CRKL determined IC50IM and IC50DAS were determined via incubating patient MNCs with the indicated concentrations of a) dasatinib and b) imatinib in the absence and presence of three ABCC6 inhibitors: pantoprazole, indomethacin, probenecid. CRKL western blotting was performed to determine the concentration of imatinib or dasatinib required for 50% Bcr-Abl kinase inhibition (IC50). The western blots depict one patient and are representative of typical results. DAS = dasatinib; IM = imatinib; PP = pantoprazole; Indo = Indomethacin; Pro = probenecid. (TIF) [file pone.0192180.s005.tif]

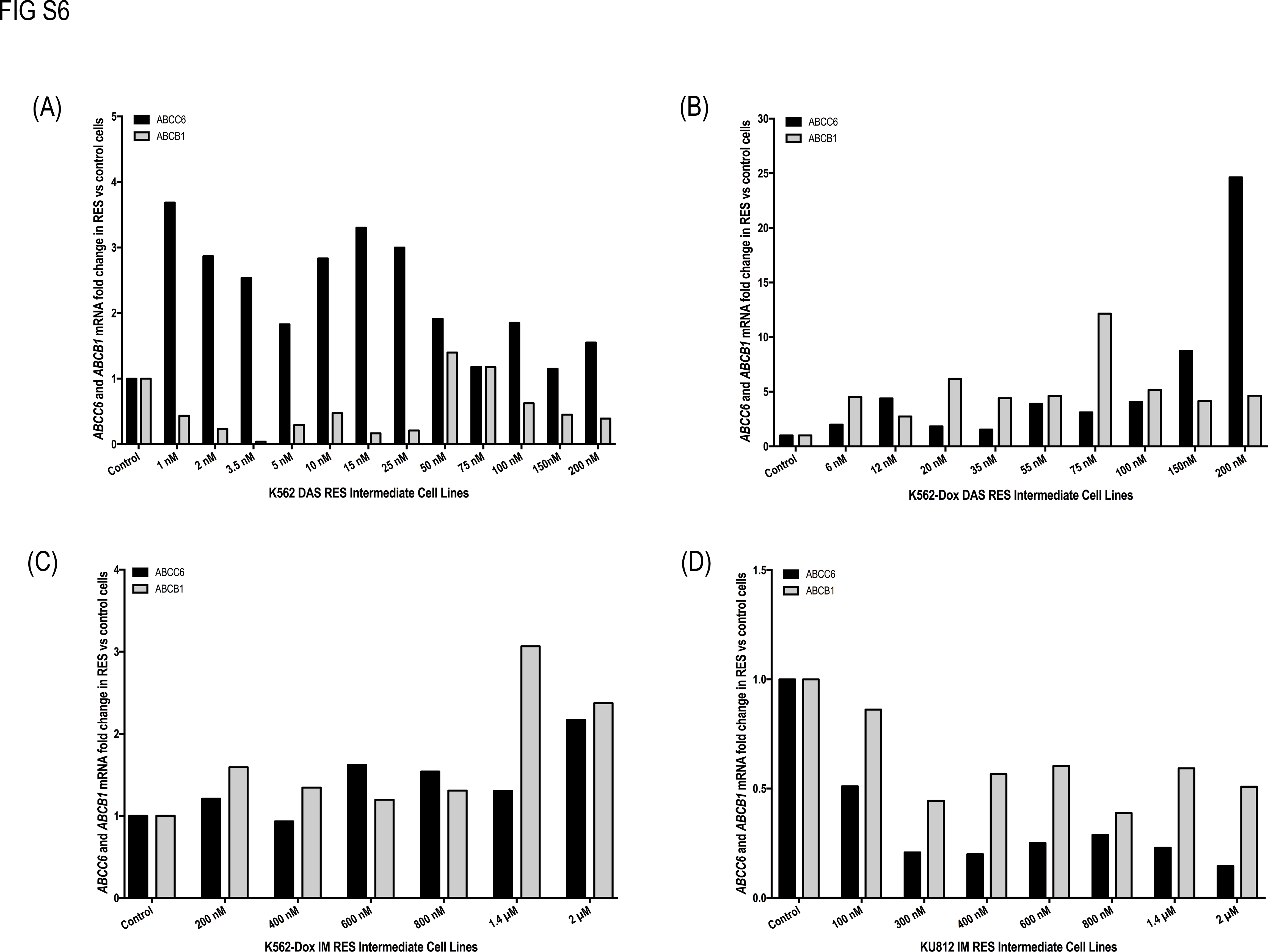

Supplement: S6 Fig — Expression levels of ABCC6 and ABCB1 mRNA were assessed in a) K562 b-c) K562-Dox cells and c) KU812 gradually made resistant to a-b) dasatinib or c-d) imatinib by exposure to increasing concentrations of TKI over time. ABCC6 and ABCB1 levels were normalized to the housekeeping gene GUSB and fold change in resistance intermediates calculated relative to control cells (control cell fold change was set at 1). The mRNA expression represents a single experiment performed in triplicate. DAS = dasatinib; IM = imatinib; RES = resistant. (TIF) [file pone.0192180.s006.tif]
