# Supplementary material for: Correction: ABCC6 plays a significant role in the transport of nilotinib and dasatinib, and contributes to TKI resistance in vitro, in both cell lines and primary patient mononuclear cells
Source: PLoS One. 2018 Aug 31;13(8):e0203583. doi: 10.1371/journal.pone.0203583 (PMC6118391; doi:10.1371/journal.pone.0203583)
Supplement: S1 Table — (DOCX) [file pone.0203583.s001.docx]

**S1 Table: Summary of inhibitors used in this study and the corresponding cellular transporters upon which they act**

|  | **Inhibitor** | | | | | | | | | | | | | | | |
| --- | --- | --- | --- | --- | --- | --- | --- | --- | --- | --- | --- | --- | --- | --- | --- | --- |
|  | PSC-833 | | | Verapamil | | | | Pantoprazole | | | Indomethacin | | | Probenecid | | |
| **Transporter** | Ref | [Inhibitor] | % Inhibition | Ref | [Inhibitor] | % Inhibition | Ref | | [Inhibitor] | % Inhibition | Ref | [Inhibitor] | % Inhibition | Ref | [Inhibitor] | % Inhibition |
| Oct-1 |  |  |  | [[1](#_ENREF_1)]  [[2](#_ENREF_2)] | 50 μM  100 μM | 69–100%  80% |  | |  |  |  |  |  |  |  |  |
| ABCB1 | [[3](#_ENREF_3)]  [[4](#_ENREF_4)]  [[5](#_ENREF_5)] | 10 μM  41 μM  5 μM | 75%  100%  67% | [[5](#_ENREF_5)]  [[6](#_ENREF_6)]  [[7](#_ENREF_7)]  [[8](#_ENREF_8)] | 20 μM  100 μM  234 μM  100 μM | 30%  70%  50%  51% | [[9](#_ENREF_9)]  [[10](#_ENREF_10)] | | 290 μM  250 μM | 50%  ~75% |  |  |  |  |  |  |
| ABCB4 | [[5](#_ENREF_5)] | 5 μM | 62% | [[5](#_ENREF_5)] | 20 μM | 42% |  | |  |  |  |  |  |  |  |  |
| ABCC1 | [[11](#_ENREF_11)] | 27 μM | 50% | [[12](#_ENREF_12)] | 8 μM | 36% |  | |  |  | [[13](#_ENREF_13)]  [[14](#_ENREF_14)] | 10 μM  100 μM | 29%  25% | [[13](#_ENREF_13)]  [[15](#_ENREF_15)]  [[14](#_ENREF_14)]  [[16](#_ENREF_16)] | 1 mM  5 mM  1 mM  1 mM | 29%  65%  90%  95% |
| ABCC2 |  |  |  |  |  |  |  | |  |  | [[14](#_ENREF_14)]  [[16](#_ENREF_16)] | 100 μM  600 μM | 5%  50% | [[16](#_ENREF_16)]  [[17](#_ENREF_17)]  [[18](#_ENREF_18)]  [[14](#_ENREF_14)]  [[19](#_ENREF_19)] | 1 mM  1 mM  260 μM  1 mM  1 mM | 25%  90%  60%  78%  90% |
| ABCC3 |  |  |  | [[20](#_ENREF_20)] | 111 μM | 13.1% |  | |  |  |  |  |  | [[18](#_ENREF_18)]  [[20](#_ENREF_20)] | 260 μM  333 μM | 70%  65% |
| ABCC4 | [[21](#_ENREF_21)] | 10 μM | 44% | [[21](#_ENREF_21)]  [[22](#_ENREF_22)] | 30 μM  25 μM | 46%  39% |  | |  |  | [[22](#_ENREF_22)]  [[23](#_ENREF_23)]  [[19](#_ENREF_19)] | 50 μM  50 μM  100 μM | 26%  89%  95% | [[24](#_ENREF_24)]  [[21](#_ENREF_21)]  [[25](#_ENREF_25)]  [[26](#_ENREF_26)]  [[19](#_ENREF_19)] | 100 μM  300 μM  1 mM  1 mM  1 mM | 48%  51%  90%  40%  80% |
| ABCC5 |  |  |  |  |  |  |  | |  |  |  |  |  | [[27](#_ENREF_27)]  [[26](#_ENREF_26)] | 50 μM  1 mM | 68%  >80% |
| ABCC6 |  |  |  |  |  |  | [[9](#_ENREF_9)]  [[9](#_ENREF_9)] | | 138 μM*  145 μM^ | 69%  80% | [[14](#_ENREF_14)] | 100 μM | 83% | [[14](#_ENREF_14)] | 1 mM | 30% |
| ABCC11 |  |  |  |  |  |  |  | |  |  | [[28](#_ENREF_28)] | 10 μM | 36% | [[28](#_ENREF_28)] | 10 μM | 24% |
| ABCG2 |  |  |  | [[29](#_ENREF_29)]  [[30](#_ENREF_30)] | 100 μM  153 μM | 21%  90% | [[31](#_ENREF_31)]  [[32](#_ENREF_32)]  [[33](#_ENREF_33)] | | 100 μM  100 μM  250 μM | 55%  95%  90% |  |  |  |  |  |  |

Results from *omeprazole and ^esomeprazole. Manuscript reported that pantoprazole demonstrated similar results but data was not shown.

## Supplementary references

1. Ahlin G, Chen L, Lazorova L, Chen Y, Ianculescu AG, Davis RL, et al. Genotype-dependent effects of inhibitors of the organic cation transporter, OCT1: predictions of metformin interactions. Pharmacogenomics J. 2011;11(6):400-11. Epub 2010/06/23. doi: 10.1038/tpj.2010.54. PubMed PMID: 20567254.

2. Zhang L, Schaner ME, Giacomini KM. Functional characterization of an organic cation transporter (hOCT1) in a transiently transfected human cell line (HeLa). J Pharmacol Exp Ther. 1998;286(1):354-61. Epub 1998/07/10. PubMed PMID: 9655880.

3. Choo EF, Leake B, Wandel C, Imamura H, Wood AJ, Wilkinson GR, et al. Pharmacological inhibition of P-glycoprotein transport enhances the distribution of HIV-1 protease inhibitors into brain and testes. Drug Metab Dispos. 2000;28(6):655-60. Epub 2000/05/23. PubMed PMID: 10820137.

4. Mayer U, Wagenaar E, Dorobek B, Beijnen JH, Borst P, Schinkel AH. Full blockade of intestinal P-glycoprotein and extensive inhibition of blood-brain barrier P-glycoprotein by oral treatment of mice with PSC833. J Clin Invest. 1997;100(10):2430-6. Epub 1997/11/20. doi: 10.1172/JCI119784. PubMed PMID: 9366556; PubMed Central PMCID: PMC508442.

5. Smith AJ, van Helvoort A, van Meer G, Szabo K, Welker E, Szakacs G, et al. MDR3 P-glycoprotein, a phosphatidylcholine translocase, transports several cytotoxic drugs and directly interacts with drugs as judged by interference with nucleotide trapping. J Biol Chem. 2000;275(31):23530-9. Epub 2000/08/05. doi: 10.1074/jbc.M909002199. PubMed PMID: 10918072.

6. Rautio J, Humphreys JE, Webster LO, Balakrishnan A, Keogh JP, Kunta JR, et al. In vitro p-glycoprotein inhibition assays for assessment of clinical drug interaction potential of new drug candidates: a recommendation for probe substrates. Drug Metab Dispos. 2006;34(5):786-92. Epub 2006/02/04. doi: dmd.105.008615 [pii]

10.1124/dmd.105.008615. PubMed PMID: 16455806.

7. Luo FR, Paranjpe PV, Guo A, Rubin E, Sinko P. Intestinal transport of irinotecan in Caco-2 cells and MDCK II cells overexpressing efflux transporters Pgp, cMOAT, and MRP1. Drug Metab Dispos. 2002;30(7):763-70. Epub 2002/06/18. PubMed PMID: 12065434.

8. Ozvegy C, Litman T, Szakacs G, Nagy Z, Bates S, Varadi A, et al. Functional characterization of the human multidrug transporter, ABCG2, expressed in insect cells. Biochem Biophys Res Commun. 2001;285(1):111-7. Epub 2001/07/05. doi: 10.1006/bbrc.2001.5130

S0006-291X(01)95130-7 [pii]. PubMed PMID: 11437380.

9. Luciani F, Spada M, De Milito A, Molinari A, Rivoltini L, Montinaro A, et al. Effect of proton pump inhibitor pretreatment on resistance of solid tumors to cytotoxic drugs. J Natl Cancer Inst. 2004;96(22):1702-13. Epub 2004/11/18. doi: 10.1093/jnci/djh305. PubMed PMID: 15547183.

10. Pauli-Magnus C, Rekersbrink S, Klotz U, Fromm MF. Interaction of omeprazole, lansoprazole and pantoprazole with P-glycoprotein. Naunyn Schmiedebergs Arch Pharmacol. 2001;364(6):551-7. Epub 2002/01/05. PubMed PMID: 11770010.

11. Leier I, Jedlitschky G, Buchholz U, Cole SP, Deeley RG, Keppler D. The MRP gene encodes an ATP-dependent export pump for leukotriene C4 and structurally related conjugates. J Biol Chem. 1994;269(45):27807-10. Epub 1994/11/11. PubMed PMID: 7961706.

12. Wong IL, Chan KF, Tsang KH, Lam CY, Zhao Y, Chan TH, et al. Modulation of multidrug resistance protein 1 (MRP1/ABCC1)-mediated multidrug resistance by bivalent apigenin homodimers and their derivatives. J Med Chem. 2009;52(17):5311-22. Epub 2009/09/04. doi: 10.1021/jm900194w. PubMed PMID: 19725578.

13. Hong J, Lambert JD, Lee SH, Sinko PJ, Yang CS. Involvement of multidrug resistance-associated proteins in regulating cellular levels of (-)-epigallocatechin-3-gallate and its methyl metabolites. Biochem Biophys Res Commun. 2003;310(1):222-7. Epub 2003/09/27. PubMed PMID: 14511674.

14. Ilias A, Urban Z, Seidl TL, Le Saux O, Sinko E, Boyd CD, et al. Loss of ATP-dependent transport activity in pseudoxanthoma elasticum-associated mutants of human ABCC6 (MRP6). J Biol Chem. 2002;277(19):16860-7. Epub 2002/03/07. doi: 10.1074/jbc.M110918200

M110918200 [pii]. PubMed PMID: 11880368.

15. Issandou M, Grand-Perret T. Multidrug resistance P-glycoprotein is not involved in cholesterol esterification. Biochem Biophys Res Commun. 2000;279(2):369-77. Epub 2000/12/19. doi: 10.1006/bbrc.2000.3939. PubMed PMID: 11118294.

16. Bakos E, Evers R, Sinko E, Varadi A, Borst P, Sarkadi B. Interactions of the human multidrug resistance proteins MRP1 and MRP2 with organic anions. Mol Pharmacol. 2000;57(4):760-8. Epub 2000/03/23. PubMed PMID: 10727523.

17. Horikawa M, Kato Y, Tyson CA, Sugiyama Y. The potential for an interaction between MRP2 (ABCC2) and various therapeutic agents: probenecid as a candidate inhibitor of the biliary excretion of irinotecan metabolites. Drug Metab Pharmacokinet. 2002;17(1):23-33. Epub 2004/12/25. PubMed PMID: 15618649.

18. Zamek-Gliszczynski MJ, Xiong H, Patel NJ, Turncliff RZ, Pollack GM, Brouwer KL. Pharmacokinetics of 5 (and 6)-carboxy-2',7'-dichlorofluorescein and its diacetate promoiety in the liver. J Pharmacol Exp Ther. 2003;304(2):801-9. Epub 2003/01/23. doi: 10.1124/jpet.102.044107. PubMed PMID: 12538836.

19. Nozaki Y, Kusuhara H, Kondo T, Iwaki M, Shiroyanagi Y, Nakayama H, et al. Species difference in the inhibitory effect of nonsteroidal anti-inflammatory drugs on the uptake of methotrexate by human kidney slices. J Pharmacol Exp Ther. 2007;322(3):1162-70. Epub 2007/06/21. doi: 10.1124/jpet.107.121491. PubMed PMID: 17578901.

20. Zeng H, Chen ZS, Belinsky MG, Rea PA, Kruh GD. Transport of methotrexate (MTX) and folates by multidrug resistance protein (MRP) 3 and MRP1: effect of polyglutamylation on MTX transport. Cancer Res. 2001;61(19):7225-32. Epub 2001/10/05. PubMed PMID: 11585759.

21. Chen ZS, Lee K, Walther S, Raftogianis RB, Kuwano M, Zeng H, et al. Analysis of methotrexate and folate transport by multidrug resistance protein 4 (ABCC4): MRP4 is a component of the methotrexate efflux system. Cancer Res. 2002;62(11):3144-50. Epub 2002/05/31. PubMed PMID: 12036927.

22. Bai J, Lai L, Yeo HC, Goh BC, Tan TM. Multidrug resistance protein 4 (MRP4/ABCC4) mediates efflux of bimane-glutathione. Int J Biochem Cell Biol. 2004;36(2):247-57. Epub 2003/12/04. PubMed PMID: 14643890.

23. Reid G, Wielinga P, Zelcer N, van der Heijden I, Kuil A, de Haas M, et al. The human multidrug resistance protein MRP4 functions as a prostaglandin efflux transporter and is inhibited by nonsteroidal antiinflammatory drugs. Proc Natl Acad Sci U S A. 2003;100(16):9244-9. Epub 2003/07/02. doi: 10.1073/pnas.1033060100. PubMed PMID: 12835412; PubMed Central PMCID: PMC170903.

24. Rius M, Nies AT, Hummel-Eisenbeiss J, Jedlitschky G, Keppler D. Cotransport of reduced glutathione with bile salts by MRP4 (ABCC4) localized to the basolateral hepatocyte membrane. Hepatology. 2003;38(2):374-84. Epub 2003/07/29. doi: 10.1053/jhep.2003.50331. PubMed PMID: 12883481.

25. van Aubel RA, Smeets PH, Peters JG, Bindels RJ, Russel FG. The MRP4/ABCC4 gene encodes a novel apical organic anion transporter in human kidney proximal tubules: putative efflux pump for urinary cAMP and cGMP. Journal of the American Society of Nephrology : JASN. 2002;13(3):595-603. Epub 2002/02/22. PubMed PMID: 11856762.

26. Reid G, Wielinga P, Zelcer N, De Haas M, Van Deemter L, Wijnholds J, et al. Characterization of the transport of nucleoside analog drugs by the human multidrug resistance proteins MRP4 and MRP5. Mol Pharmacol. 2003;63(5):1094-103. Epub 2003/04/16. PubMed PMID: 12695538.

27. Jedlitschky G, Burchell B, Keppler D. The multidrug resistance protein 5 functions as an ATP-dependent export pump for cyclic nucleotides. J Biol Chem. 2000;275(39):30069-74. Epub 2000/07/13. doi: 10.1074/jbc.M005463200. PubMed PMID: 10893247.

28. Chen ZS, Guo Y, Belinsky MG, Kotova E, Kruh GD. Transport of bile acids, sulfated steroids, estradiol 17-beta-D-glucuronide, and leukotriene C4 by human multidrug resistance protein 8 (ABCC11). Mol Pharmacol. 2005;67(2):545-57. Epub 2004/11/13. doi: 10.1124/mol.104.007138. PubMed PMID: 15537867.

29. Ozvegy-Laczka C, Hegedus T, Varady G, Ujhelly O, Schuetz JD, Varadi A, et al. High-affinity interaction of tyrosine kinase inhibitors with the ABCG2 multidrug transporter. Mol Pharmacol. 2004;65(6):1485-95. Epub 2004/05/25. doi: 10.1124/mol.65.6.1485

65/6/1485 [pii]. PubMed PMID: 15155841.

30. Scharenberg CW, Harkey MA, Torok-Storb B. The ABCG2 transporter is an efficient Hoechst 33342 efflux pump and is preferentially expressed by immature human hematopoietic progenitors. Blood. 2002;99(2):507-12. Epub 2002/01/10. PubMed PMID: 11781231.

31. Breedveld P, Pluim D, Cipriani G, Wielinga P, van Tellingen O, Schinkel AH, et al. The effect of Bcrp1 (Abcg2) on the in vivo pharmacokinetics and brain penetration of imatinib mesylate (Gleevec): implications for the use of breast cancer resistance protein and P-glycoprotein inhibitors to enable the brain penetration of imatinib in patients. Cancer Res. 2005;65(7):2577-82. Epub 2005/04/05. doi: 65/7/2577 [pii]

10.1158/0008-5472.CAN-04-2416. PubMed PMID: 15805252.

32. Suzuki K, Doki K, Homma M, Tamaki H, Hori S, Ohtani H, et al. Co-administration of proton pump inhibitors delays elimination of plasma methotrexate in high-dose methotrexate therapy. Br J Clin Pharmacol. 2009;67(1):44-9. Epub 2008/12/17. doi: BCP3303 [pii]

10.1111/j.1365-2125.2008.03303.x. PubMed PMID: 19076159; PubMed Central PMCID: PMC2668083.

33. Breedveld P, Zelcer N, Pluim D, Sonmezer O, Tibben MM, Beijnen JH, et al. Mechanism of the pharmacokinetic interaction between methotrexate and benzimidazoles: potential role for breast cancer resistance protein in clinical drug-drug interactions. Cancer Res. 2004;64(16):5804-11. Epub 2004/08/18. doi: 10.1158/0008-5472.CAN-03-4062

64/16/5804 [pii]. PubMed PMID: 15313923.
