# Supplementary material for: Correction: ABCC6 plays a significant role in the transport of nilotinib and dasatinib, and contributes to TKI resistance in vitro, in both cell lines and primary patient mononuclear cells
Source: PLoS One. 2018 Aug 31;13(8):e0203583. doi: 10.1371/journal.pone.0203583 (PMC6118391; doi:10.1371/journal.pone.0203583)
Supplement: S1 File — (DOCX) [file pone.0203583.s002.docx]

# Supporting information

## Supplementary methods

### Western blotting for ABCC6

Western blotting for ABCC6 (abcam®, Cambridge, UK) was performed using the BIO-RAD Trans-Blot^®^ Turbo^TM^ Blotting System. 5×10^6^ cells were lysed in modified RIPA buffer (50 mM Tris-HCl, 1% NP-40, 0.25% Na-deoxycholate, 150 mM NaCl) containing protease inhibitors (Pefabloc, Sigma; cOmplete, Roche, Basel, Switzerland) and HALT phosphatase inhibitor (Thermo Fisher Scientific). 100 μg protein was resolved on a BIO-RAD 4-15% Criterion^TM^TGX^TM^ Precast Gel and then transferred to PVDF membrane using Midi Transfer Packs (BIO-RAD, Hercules, CA, USA) at 2.5 A for 10 min. Membranes were blocked with 5% BSA and incubated with primary antibody according to the manufacturer’s instructions prior to incubation with HRP-conjugated anti-rabbit immunoglobulin (Thermo Fisher Scientific) and detection with ECL substrate (BIO-RAD) by Chemidoc (BIO-RAD). Membranes were probed for the control protein β-actin (Sigma) following the same incubation conditions as described for ABCC6. Detected proteins were quantified using Image Studio Lite software v5.0.21 (Licor, Lincoln, NE, USA) and expression normalised to β-actin.

### Real time quantitative polymerase chain reaction (RQ-PCR)

*ABCB1* F: 5’ AGA CAT GAC CAG GTA TGC CTA T 3’

*ABCB1* R: 5’ AGC CTA TCT CCT GTC GCA TTA 3’

*ABCC6* F: 5’ ACA CTT CAA TTG GGG AGC AG 3’

*ABCC6* R: 5’ TGT TGT TCC CTG GAG TAG CC 3’

*BCR* F: 5’ CCT TCG ACG TCA ATA ACA AGG AT 3’

*BCR* R: 5’ CCT GCG ATG GCG TTC AC 3’

*GUSB* F: 5’ CTG AAC AGT CAC CGA CGA GA 3’

*GUSB* R: 5’ GAA CGC TGC ACT TTT TGG TT 3
